# *CaDHN5*, a Dehydrin Gene from Pepper, Plays an Important Role in Salt and Osmotic Stress Responses

**DOI:** 10.3390/ijms20081989

**Published:** 2019-04-23

**Authors:** Dan Luo, Xiaoming Hou, Yumeng Zhang, Yuancheng Meng, Huafeng Zhang, Suya Liu, Xinke Wang, Rugang Chen

**Affiliations:** College of Horticulture, Northwest A&F University, Yangling 712100, China; danluonwafu@163.com (D.L.); 15230286139@163.com (X.H.); Kexuanzhangyumeng@163.com (Y.Z.); YuanchengMeng07@126.com (Y.M.); 18848966687@163.com (H.Z.); YaSuLiu@126.com (S.L.); W1942399775@126.com (X.W.)

**Keywords:** *Capsicum annuum* L., *CaDHN5*, salt stress, osmotic stress, dehydrin

## Abstract

Dehydrins (*DHNs*), as a sub-family of group two late embryogenesis-abundant (LEA) proteins, have attracted considerable interest owing to their functions in enhancing abiotic stress tolerance in plants. Our previous study showed that the expression of *CaDHN5* (a dehydrin gene from pepper) is strongly induced by salt and osmotic stresses, but its function was not clear. To understand the function of *CaDHN5* in the abiotic stress responses, we produced pepper (*Capsicum annuum* L.) plants, in which *CaDHN5* expression was down-regulated using VIGS (Virus-induced Gene Silencing), and transgenic *Arabidopsis* plants overexpressing *CaDHN5*. We found that knock-down of *CaDHN5* suppressed the expression of manganese superoxide dismutase *(MnSOD*) and peroxidase (*POD*) genes. These changes caused more reactive oxygen species accumulation in the VIGS lines than control pepper plants under stress conditions. *CaDHN5*-overexpressing plants exhibited enhanced tolerance to salt and osmotic stresses as compared to the wild type and also showed increased expression of salt and osmotic stress-related genes. Interestingly, our results showed that many salt-related genes were upregulated in our transgenic *Arabidopsis* lines under salt or osmotic stress. Taken together, our results suggest that *CaDHN5* functions as a positive regulator in the salt and osmotic stress signaling pathways.

## 1. Introduction

Plants live in an open environment and cannot move from one place to another. As a result, plants are exposed to various biotic and abiotic stresses. These stresses individually, or in combination, result in huge losses in terms of growth, development, and yield, and sometimes threaten the survival of the plant. Amongst the abiotic factors, water stress is the most important [1,2]. Some earlier studies treated drought and salinity as similar stresses because plants respond in a similar manner to salt and drought stresses, and signaling mechanisms overlap [3].

Dehydrin, a highly hydrophilic plant protein, belongs to the second sub-family of the late embryogenesis developmental protein family (LEA II) [4]. The protein sequence has conserved K-segments (consisting of EKKGIMDKIKEKLPG located near the C-terminus, rich in lysine), Y-segments (consisting of T/VDEYGNP located close to the N-terminus), and S-segments (rich in serine) [5]. Dehydrins are classified into five categories: Y_n_SK_n_, K_n_, SK_n_, Y_n_K_n_, and K_n_S [6]. Every type of dehydrin has a different function. For example, SK_n_ dehydrins can not only bind phospholipids, protect enzyme stability, and prevent heat-induced degeneration, but they are also crucial for plant growth, development, and resistance to low temperature stress responses [7]. Various abiotic stresses and hormones can strongly influence expression of dehydrin [8]. Studies have shown that Y_n_SK_n_ is an alkaline or neutral protein that is highly upregulated by cold stress [9], and has a unique RRKK motif (a nuclear localization signal), which is key for the localization of Y_n_SK_n_-type dehydrins in the nucleus. Many studies have shown that there is a positive interaction between the expression of dehydrins and resistance to abiotic stresses [10]. *Cicer pinnatifidum* Y_2_K-type dehydrin *CpDHN1* and white spruce S_8_K_4_-type dehydrin *PgDHN1* were induced by methyl jasmonate (MeJA) and salicylic acid (SA) [11,12]. *CaDHN5* belongs to the YSK_2_ category of dehydrins. It is a neutral or basic protein that can be induced by osmotic or salt stresses, and exogenous abscisic acid (ABA), as shown in our previous study [13].

In recent years, many studies have explored the functional importance of dehydrin in plant stress resilience [14,15,16,17,18,19,20]. In addition, since the expression of various dehydrin genes can be induced by exogenous ABA treatment, dehydrins are also considered as ABA-responsive proteins (ABR) [14]. In tomato plants overexpressing dehydrin gene, drought resistance was enhanced without influencing tomato growth traits [15]. In barley, cold acclimation was due to faster *DHN5* accumulation rates in the winter lines compared to that of spring lines [16]. In *Physcomitrella*, under salt and mannitol stresses, the expression of *PpDHNA* and *PpDHNB* were strongly up-regulated [17]. Studies in transgenic *Arabidopsis thaliana* plants showed that overexpression of *AmDHN* (*Ammopiptanthus mongolicus* dehydrin) improved osmotic stress tolerance and drought resistance [18]. *MusaDHN-1*, an SK_3_-type dehydrin gene in banana, contributes positively towards drought and salt stress tolerance, and responses to abscisic acid, ethylene, and methyl jasmonate [19]. Similarly, in *Boea crassifolia,* overexpression of YNSK_2_-type dehydrin, *BcDh2* enhanced tolerance to mechanical stress, mediated by salicylic acid and jasmonic acid [20].

In a previous study, it was found that specific *DHNs* in pepper are differentially induced in response to different stresses [21]. Among the seven *Capsicum annuum* dehydrin genes, *CaDHN5* was significantly up-regulated under salt and osmotic stress treatments [13]. Therefore, in this study, we further explored the relationship between *CaDHN5* and salt and osmotic stresses via overexpressing and gene silencing techniques. The results showed that *CaDHN5*-silenced pepper plants were less tolerant to salt and osmotic stress, while *CaDHN5*-overexpressing *Arabidopsis* plants showed significantly increased tolerance to these stresses. These results suggest that *CaDHN5* functions as a positive regulator in salt and osmotic stress signaling pathways.

## 2. Results

### 2.1. Analysis of Silencing Efficiency of CaDHN5 in Pepper

**Figure 1:**
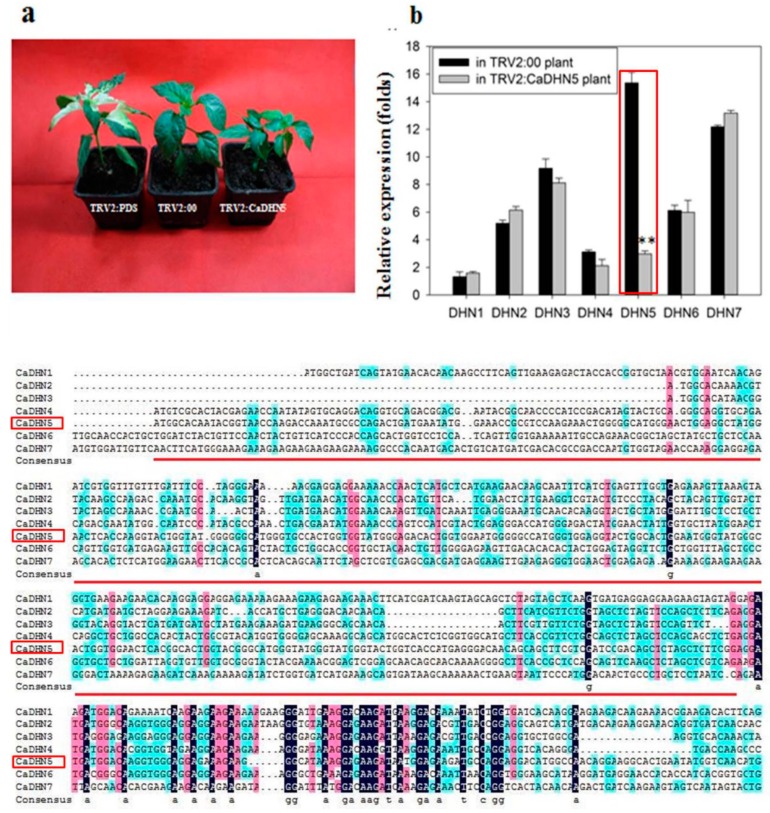
Phenotypes of *CaDHN5*-silenced plants and detection of gene silencing efficiency. (**a**) The phenotypes of silenced pepper plants (about four weeks after injection); (**b**) The relative expression of *CaDHN5* in silenced pepper plants and silencing sequence analysis of *CaDHN5*. The red frame is expression of *CaDHN5* in silenced and control plants. The black areas represent homology level 100%. The pink areas represent a level of homology greater than or equal to 75%. The blue areas represent a level of homology greater than or equal to 50%. The underlined part is a sequence of silencing. The results are the means ± standard deviation (S.D.), replicated three times. The means were compared using Student’s test. Note: * indicates significant differences compared with the control at *p* < 0.01.

Virus-Induced Gene Silencing (VIGS) technique was used to investigate the function of *CaDHN5* under salt and osmotic stresses [22,23]. About 310 bp specific sequences from *CaDHN5* were used to construct the vector pTRV2:*CaDHN5*. Phytoene desaturase (*PDS*) was used as a marker of gene silencing, due to its ability to induce a bleached phenotype after successfully silencing plants [22]. Approximately four weeks after the induction of TRV-mediated gene silencing, pepper plants induced with TRV2:*CaPDS* began to show an albino phenotype, while plants with the TRV2 empty vector and TRV2:*CaDHN5* showed no difference in phenotype (Figure 1a). We detected the expression level of the other six genes within the dehydrin family, and found that only *DHN5* expression level was down-regulated (Figure 1b). From the result of *CaDHN5* expression, it can be seen that the expression decreased by about 80% in the fourth week after induction in *CaDHN5*-silenced plants.

### 2.2. Influence of Silencing CaDHN5 on Tolerance of Salt and Osmotic Stresses in Pepper

**Figure 2:**
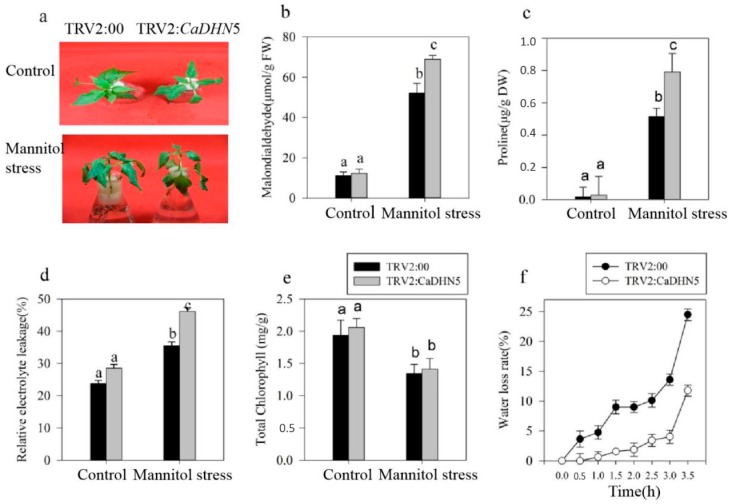
Effects of osmotic stress on plant phenotypes. (**a**) The phenotype of *CaDHN5*-silenced pepper plants under osmotic stress; (**b**) MDA content; (**c**) proline levels; (**d**) relative electrolytic leakage; (**e**) total Chlorophyll content; (**f**) water loss. Data that are significantly different are indicated with letters above the error bars (±S.D.). The different letters with the bars indicate significant differences as determined using Tukey HSD’s multiple range tests (*p* < 0.05).

To investigate the effect of *CaDHN5* silencing on the osmotic tolerance of pepper plants, control and *CaDHN5*-silenced plants were treated with 250 mM mannitol under continuous lighting conditions for three days. *CaDHN5*-silenced plants wilted considerably more than control plants, and some leaves became yellow after three days in mannitol-treated plants (Figure 2a).

In order to compare the differences between *CaDHN5*-silenced and control plants under mannitol treatment, we measured relative electrolyte leakage, rate of water loss, malondialdehyde (MDA), and chlorophyll content of these plants (Figure 2b–f). Following mannitol treatment, MDA levels in the control plants increased about four-fold, compared to control water treated plants, while in *CaDHN5*-silenced pepper plants, MDA levels increased by about six-fold (Figure 2b). After mannitol treatment, the proline content of silenced pepper plants increased two times as much as that seen in control plants (Figure 2c).

The rate of water loss and relative electrolyte leakage are indicators of the degree of membrane injury [24]. As can be seen from the relative electrolyte leakage measurements, the degree of membrane injury of pepper plants was significantly higher under mannitol treatment compared to controls (Figure 2d). Under 250 mM mannitol treatment, total chlorophyll content in control and silenced plants were both significantly decreased, and the difference between control and silenced plants was not significant (Figure 2e). In silenced plants, the rate of water loss increased, and the rate of water loss was three-fold lower than control plants after mannitol treatment (Figure 2f).

**Figure 3:**
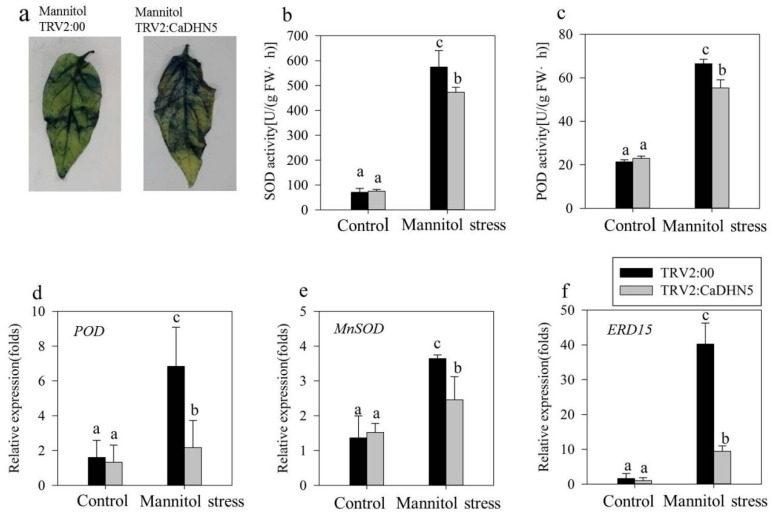
Determination of oxidative stress resistance and stress-related gene expression in mannitol treatment. (**a**) Results of pepper plants stained with NBT under mannitol treatment; (**b**) SOD activity under osmotic stress; (**c**) POD activity under mannitol treatment; (**d**) relative expression of *POD* under mannitol treatment; (**e**) *MnSOD* relative expression under mannitol treatment; (**f**) relative expression of *ERD15* under mannitol treatment. Data that are significantly different are indicated with letters above the error bars (±S.D.). The different letters with the bars indicate significant differences as determined using Tukey HSD’s multiple range tests (*p* < 0.05).

In normal condition the activities of superoxide dismutase (SOD) and peroxidase (POD) were not significantly different between control and silenced plants. However, after mannitol treatment, the activities of SOD and POD increased to scavenge superoxide anions and H_2_O_2_ produced in the plant. Therefore, the enzyme activities could reflect the ability of the plant to scavenge superoxide anions and H_2_O_2_. From Figure 3b,c, it can be clearly seen that in the *CaDHN5* silenced plant, the increase in enzyme activity was significantly lower than that of the control plant. The staining results of NBT also showed that after gene silencing, more superoxide anions accumulated in the leaves (Figure 3a).

We also analyzed the expression of the stress and antioxidant system-related genes (*MnSOD*, *POD*, and *ERD15* [23]) in control and silenced lines. There was no significant difference in the expression of *POD* between control and silenced lines before treatment. After mannitol treatment, the expression of *POD* in control and silenced lines both increased, but in silenced plants it only increased two-fold, while in control plants this increase was four-fold (Figure 3d). A similar result was found for the expression of *MnSOD*. After *CaDHN5* silencing, the increased expression of *MnSOD* in silenced plants was only half compared to control plants (Figure 3e). In addition, the expression of *ERD15* was significantly higher in control plants treated with mannitol, while in silenced plants, increased expression of *ERD15* was significantly higher than control but less than mannitol-treated silenced pepper plants (Figure 3f).

**Figure 4:**
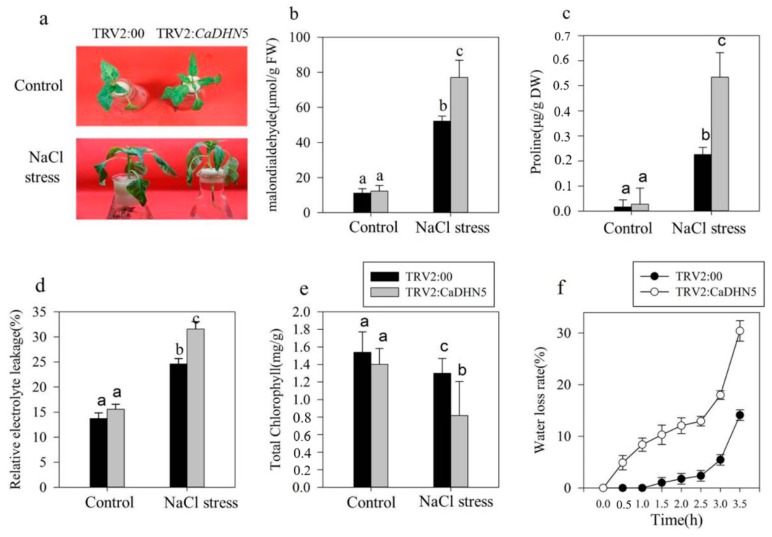
Effects of salt stress on plant phenotypes. (**a**) The phenotype of *CaDHN5*-silenced pepper plants under NaCl treatment; (**b**) MDA content; (**c**) proline levels; (**d**) relative electrolytic leakage; (**e**) total chlorophyll content; (**f**) water loss. Data that are significantly different are indicated with letters above the error bars (±S.D.). The different letters with the bars indicate significant differences as determined using Tukey HSD’s multiple range tests (*p* < 0.05).

In order to investigate the effect of silencing *CaDHN5* on salt stress in pepper, we measured the same physiological indices as those measured for mannitol stress, and performed the same analysis. Silenced and control plants were treated with 250 mM NaCl solution. Regarding the phenotype, the wilting conditions of silenced pepper plants were more evident under NaCl treatment (Figure 4a). Under normal conditions, there was no significant difference in the MDA content between control and silenced plants. However, after NaCl treatment, MDA levels in both control and silenced plants increased significantly; this increase in silenced plants was one and a half times more than that in control plants. Therefore, it appears that *CaDHN5*-silenced pepper plants experienced more serious membrane lipid peroxidation than control plants (Figure 4b). After NaCl treatment, the proline content of silenced pepper plants increased five-fold compared to control plants (Figure 4c). The chlorophyll content of *CaDHN5*-silenced pepper plants decreased more rapidly (Figure 4e). In silenced plants under NaCl treatment, the rate of water loss was four-fold faster than control plants (Figure 4f).

**Figure 5:**
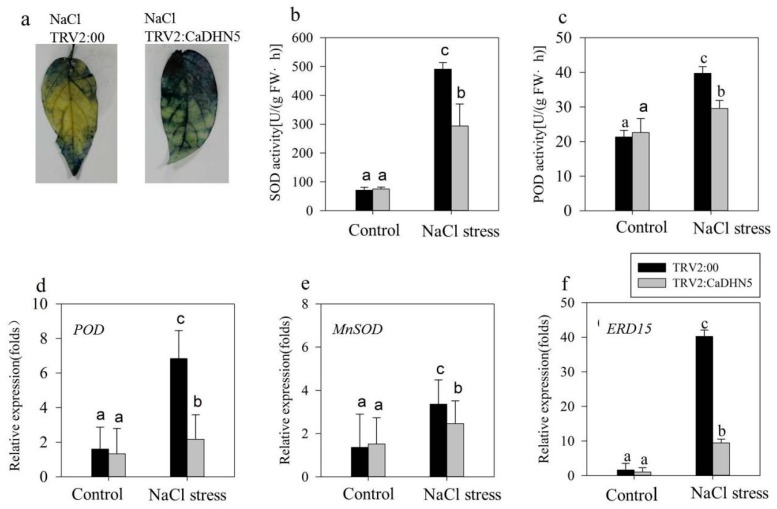
Determination of oxidative stress resistance and stress-related gene expression in NaCl treatment. (**a**) Results of NBT-stained pepper plants under NaCl treatment; (**b**) SOD activity under NaCl stress; (**c**) POD activity under NaCl treatment; (**d**) *POD* relative expression under NaCl treatment; € *MnSOD* relative expression under NaCl treatment; (**f**) *ERD15* relative expression under NaCl treatment. Data significantly different are indicated with letters above the error bars (±S.D.). The different letters with the bars indicate significant differences as determined using Tukey HSD’s multiple range tests (*p* < 0.05).

The activities of SOD and POD were measured under NaCl treatment. Results show that the activities increased in both silenced and control plants following NaCl treatment, but were slightly lowered in silenced plants compared to control pepper plants. These results suggested that the ability of plants to remove superoxide anions and H_2_O_2_ decreased slightly in silenced plants (Figure 5b,c). The NBT staining data reflected these observations (Figure 5a). In silenced plants treated with NaCl, NBT-stained leaves were more than that in the control lines. These data suggested that more superoxide anion accumulation occurred in silenced plants. We monitored the expression of POD and ERD15 (Figure 5d,f), which showed a significant increase in control plants treated with NaCl. MnSOD also exhibited a significant increase in expression, although this increase was less than that of POD and ERD15.

### 2.3. Analysis of CaDHN5-Overexpression Arabidopsis

**Figure 6:**
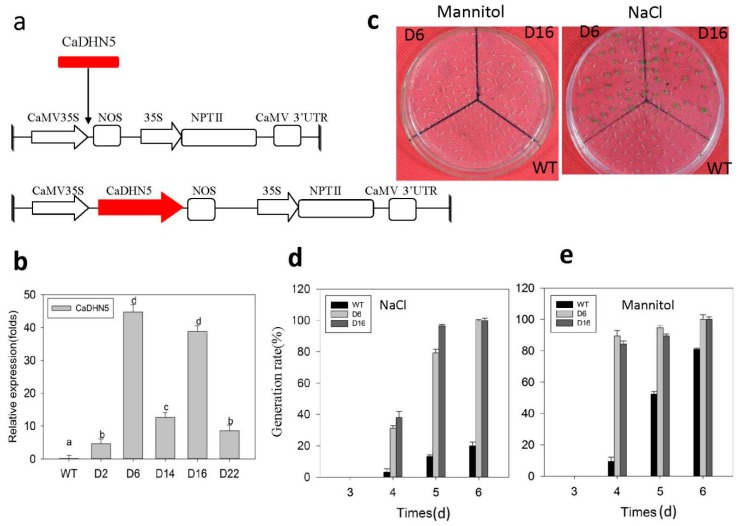
Assay of the transgenic *CaDHN5*-overexpressing lines. (**a**) Schematic representation of the pVBG2307:*CaDHN5* construct; (**b**) qRT-PCR analysis of *CaDHN5* expression in *Arabidopsis* transgenic lines (D2, D6, D14, D16, D22), with WT as control; (**c**) the phenotype of seed germination in wild type and two transgenic *Arabidopsis* plants (D6 and D16) subjected to salt (NaCl) and osmotic (mannitol) stress for five days; (**d**,**e**) seed germination rates of different lines subjected to salt (NaCl) and osmotic (mannitol) stress. Data significantly that are different are indicated with letters above the error bars (±S.D.). The different letters with the bars indicate significant differences as determined using Tukey HSD’s multiple range tests (*p* < 0.05).

We constructed the overexpression vector pVBG2307:*CaDHN5*. The schematic diagram of the vector is shown in Figure 6a. After *Agrobacterium*-mediated transformation, the expression level of *CaDHN5* was estimated by qRT-PCR (Figure 6b). Expression of *CaDHN5* was higher in the lines D6 and D16 than other lines, so their homozygous T3 generation plants were chosen for further physiological analyses.

### 2.4. Seed Germination under Osmotic and Salt Stress Conditions

Transgenic *Arabidopsis* seeds were germinated on MS/2 agar medium containing 200 mM NaCl or mannitol solutions, and the germination rate was calculated (Figure 6c). Under NaCl treatment, at five days, almost all transgenic-seeds were germinated. However, only about 13% of WT seeds germinated (Figure 6d). Meanwhile, after six days, almost all transgenic seeds were germinated, while only 20% of the WT seeds were germinated. A similar trend was followed in the presence of mannitol, where the transgenic lines showed better germination compared to the WT seeds. At five days, almost all transgenic-seeds were germinated, while only 13% of WT seeds were germinated. In the following days, the germination rate of WT gradually increased, eventually reaching 80%, and transgenic seeds reached 100% (Figure 6e). These data show that the transgenic D6 and D16 lines displayed a better rate of seed germination than WT under salinity or osmotic stress.

### 2.5. Increased Tolerance of CaDHN5-Overexpressing Transgenic Arabidopsis Plants towards Salt and Osmotic Stresses

**Figure 7:**
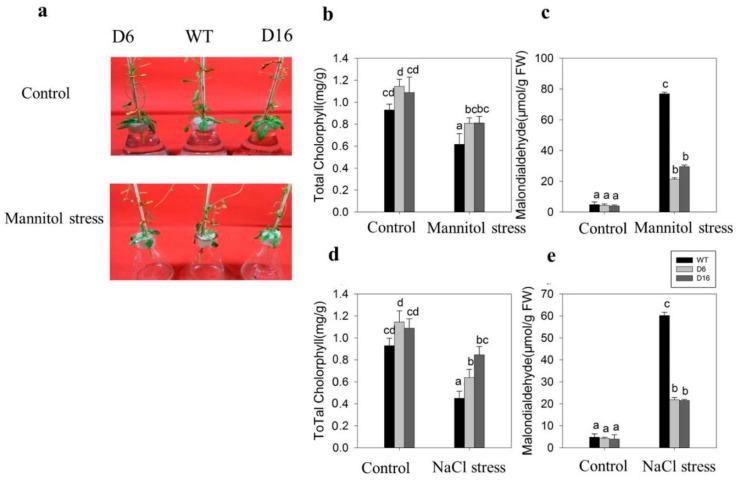
Related-physiological indices of *Arabidopsis thaliana* under salt and osmotic stress treatments. (**a**) The phenotypes of wild type (WT) and *CaDHN5*-overexpressing transgenic plants (D6 and D16) under mannitol treatment; (**b**) effects of mannitol treatment on total chlorophyll in transgenic *Arabidopsis* plants; (**c**) effects of mannitol treatment chlorophyll content in transgenic *Arabidopsis* plants; (**d**) effects of NaCl treatment on MDA content in transgenic *Arabidopsis* plants; (**e**) effects of NaCl treatment on total chlorophyll content in transgenic *Arabidopsis* plants. Data that are significantly different are indicated with letters above the error bars (±S.D.). The different letters with the bars indicate significant differences as determined using Tukey HSD’s multiple range tests (*p* < 0.05).

After three days treatment with 250 mM NaCl or mannitol, we observed the phenotype of *CaDHN5*-overexpressing transgenic *Arabidopsis* plants and measured physiological parameters (Figure 7).With mannitol treatment, the phenotypes of all *Arabidopsis thaliana* plants showed varying degrees of water loss, which occurred in all *Arabidopsis thaliana* plants, and the whole plants became brittle. After three days of mannitol treatment, WT leaves had suffered severe water loss and became brittle, while the leaves of transgenic plants remained moist (Figure 7a).

**Figure 8:**
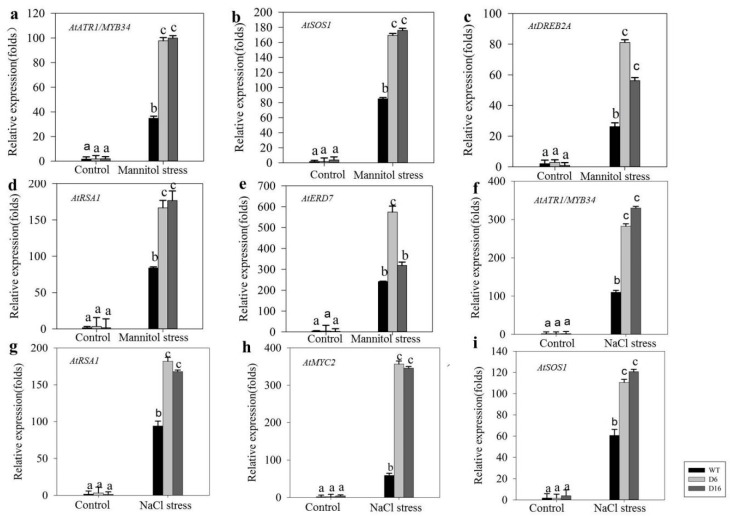
Expression of salt and osmotic related-genes in wild-type and transgenic plants. (**a**,**f**) *AtATR/MYB34* from *Arabidopsis* under mannitol and NaCl treanment; (**b**,**i**) *AtSOS1* from *Arabidopsis* under mannitol and NaCl treatments; (**c**) *AtDREB2A* from *Arabidopsis* under NaCl treatments; (**d**,**g**) *AtRSA1* from *Arabidopsis* under mannitol and NaCl treatments; (**e**) *AtERD7* from Arabidopsis under mannitol treatments; (**h**) *AtMYC2* from *Arabidopsis* under NaCl treanment. Data that are significantly different are indicated with letters above the error bars (±S.D.). The different letters with the bars indicate significant differences as determined using Tukey HSD’s multiple range tests (*p* < 0.05).

As can be seen from the content of MDA and chlorophyll (Figure 8b,c), the injury of wild-type plants was more serious under 250 mM mannitol treatment. Due to the influence of mannitol and salt, the chlorophyll content decreased to a similar extent in both WT and transgenic lines compared to controls (Figure 7b). The MDA content in WT following mannitol treatment increased by 15-fold compared to the control conditions, while in two transgenic lines D6 and D16, the increase was recorded to be about five-fold compared to controls (Figure 7c). After 250 mM NaCl treatment, the MDA content in WT increased by 12-fold, whereas in the two transgenic lines this increase was only about four-fold.

Based on previous research, we also selected several stress-related genes, *AtDREB2A, AtDREB2B* [25], *AtERD7*, and *AtMYC2* [26], to assess responses to osmotic stress, and *AtATR1/MYB34* [26]*, AtSOS1* [25]*, AtRITF1* [27]*,* and *AtRSA1* for responses to salt stress in *Arabidopsis*. The relative expression levels of the above-mentioned genes were measured in WT and *CaDHN5*-overexpressing plants under stress and mannitol stresses (Figure 8). Only genes with significant changes are shown in Figure 8. *AtATR1/MYB34*, *AtSOS1*, and *AtRSA1* were up-regulated in both osmotic and salt stresses.

## 3. Discussion

The LEA family of proteins were originally thought to be induced during seed maturation and drying [28]. In this study, *CaDHN5* cDNA was isolated from pepper leaves. Our results indicate *CaDHN5* shows a strong response to salt and osmotic stresses. In the experiments, NaCl and mannitol treatment were used to simulate salt and osmotic stresses. *CaDHN5* silenced and overexpressing transgenic plants were used to verify the function of *CaDHN5*. Silenced pepper plants were more sensitive to the effects of high salt and osmotic stresses, and *CaDHN5*-over-expressed plants were more tolerant than the WT plants.

We silenced *CaDHN5* in the pepper plant cultivar “P70”. We first examined expression of *CaDHN5* in silenced pepper plants to ensure that subsequent experiments were carried out on the premise of successful gene silencing. Plant tolerance to stress is closely related to some physiological indices. It is well known that plants with strong stress tolerance usually have higher chlorophyll content and lower content of electrolyte leakage, proline, and MDA under stress situations. Under salt and osmotic stress conditions, the different trends in the decrease or increase of chlorophyll content, MDA, and conductivity suggest that *CaDHN5* may be involved in salt and osmotic stress responses. Meanwhile, these results indicated that the membrane damage and leaf senescence of the silenced *CaDHN5* pepper plants were higher under salt and osmotic stresses. Other studies in different plants have shown similar results [29,30]. Under salt stress and osmotic stresses, many *DHNs* were up-regulated in transgenic *Arabidopsis* plants, which showed high tolerance to these stresses [29]. It has also been found that the barley dehydrin *DHN3* responds to various stresses [30]. *POD* and *MnSOD* are important genes that function in the process of scavenging ROS. We found that when *CaDHN5* was silenced in pepper, the expression levels of these two genes were significantly lower than those of the control plants under salt and osmotic stress. This indicates that *CaDHN5* positively regulates the expression of these genes. In addition, results of enzyme activity and staining with NBT indicated that gene-silenced plants had higher levels of superoxide anion. *CaDHN5*-silenced pepper plants had lower tolerance to salt and osmotic stresses.

Further, we generated *CaDHN5* over-expressing transgenic *Arabidopsis*. Under high salinity and osmotic stress, it was found that when *CaDHN5* was overexpressed in *Arabidopsis*, it resulted in increased tolerance to salt and osmotic stress. Previous reports have described the increased anti-stress ability of different LEA genes in various plants, such as rice, wheat, and *Arabidopsis* [31,32]. The *MusaDHN1* gene of banana is not only induced by drought, salt, cold, oxidation, and heavy metal stress, but can also be induced by abscisic acid, ethylene, and methyl jasmonate [19]. In this study, *CaDHN5* transgenic lines are more tolerant under high concentrations of NaCl and mannitol. Transgenic seeds germinate rapidly under 200 mM mannitol compared to WT. Studies have shown that the dehydrin in Chinese cabbage has a similar function [33]. When we studied the influence of *CaDHN5* on salt stress tolerance, we found significant differences in MDA content between WT and transgenic plants. Meanwhile, overexpression of *CaDHN5* in *Arabidopsis* resulted in decreasing Chlorophyll degradation under NaCl treatment, but had no significant effect under osmotic stress. This could have resulted from the low accumulation of MDA in transgenic lines. In salt and osmotic stresses, the germination rates of *CaDHN5*-overexpressing *Arabidopsis* plants in the presence of NaCl and mannitol were significantly higher than those of WT plants. Monitoring the expression of other salts and osmotic stress-related genes showed that when *CaDHN5* was overexpressed, the expression levels of these stress-related genes also increased to varying degrees. Other studies have also shown similar results. When transgenic *Arabidopsis thaliana* transformed with wheat *TaDHN1* and *TaDHN3* genes were treated with salt and mannitol, the transgenic plants grew better and the root lengths were longer than wild type [34]. *HbDHN1*, *HbDHN2* were also transformed into *Arabidopsis thaliana*. *Arabidopsis thaliana* was transformed with *HbDHN1*, and *HbDHN2* reduced electrolyte leakage of cells and accumulation of ROS by increasing the SOD and POD activity, thereby resisting salt and osmotic stress [35]. In fact, among the expression of the eight related genes that we recorded, five genes (*AtATR1/MYB34, AtSOS1, AtDERB2A, AtRSA1,* and *AtERD7*) were up-regulated under osmotic stress, and four (*AtATR1/MYB34, AtRSA1, AtMYC2,* and *AtSOS1*) were up-regulated under salt stress. As mentioned earlier, signaling pathways in response to salt and osmotic stresses overlap [3]. The expression of *AtSOS1*, which encoded a plasma membrane Na^+^/H^+^ antiporter essential for salt tolerance. [27], was significantly increased in transgenic lines, both under salt and osmotic stresses. The expression of the transcription factor *AtDREB2A* in the ABA signaling pathway was also significantly increased. *AtRSA1* and *AtRITF1* are interacting genes that not only participate in the regulation of the transcription of several genes in the ROS scavenging system, but also regulate the expression of *AtSOS1*. It is worth noting that although the expression of *AtRSA1* gene was increased under salt and osmotic stresses, the interacting partners of *AtRSA1* and *AtRITF1* were only up-regulated under salt treatment.

## 4. Materials and Methods

### 4.1. Plant Materials, Growth Conditions

Seeds (wild-type: Columbia ecotype) and pepper (*Capsicum annuum* L.) cultivar “P70” were used in the current work, which were provided by Vegetable Plant Biotechnology and Germplasm Innovation laboratory, Northwest A&F University-China. The *Arabidopsis thaliana* seeds were treated as per Brini’s method [32]. The pepper seedlings were cultured in a growth chamber by maintaining them in 16 h/8 h light/dark at 25 °C/20 °C [23]. The control plants were grown in the same environment and treated with corresponding solvents.

### 4.2. Isolation CaDHN5

According to the full-length CaDHN5 ORF sequence (GenBank accession No.: XM016705201), forward and reverse primers were designed as 5’-AGGAGATGGCACAATACGGT-3’AND5’-ATCCTTTGTTTTCATTTTCAGC-3’, respectively. PCR products were cloned into the pMD19-T vector (TaKaRa, Dalian, China) and sequenced (Xi’an AuGCT Biotechnologies Co. Xi’an, China).

### 4.3. Silencing Efficiency Analysis of CaDHN5 in Pepper

The pTRV2: *CaDHN5* construct was engineered to include a 310 bp sequence in *CaDHN5* cloned from a pepper cDNA template, using the forward primers 5’-ATGGCACAATACGGTAACC-3’and the reverse primers 5’-CCGAAGAGCTAGAGCTGTC-3’. The recombinant plasmid pTRV2: *CaDHN5* was constructed by combining CaDHN5 and pTRV2. *Agrobacterium tumefaciens* GV3101 containing pTRV2:*CaDHN5* was injected into pepper plants after combining GV3101 with pTRV1, and plants were grown as described previously. Fifty plants were used for the silencing assay [21].

### 4.4. Generation of Transgenic Arabidopsis Plants

The vector pVBG2307:*CaDHN5* contains the kanamycin resistance gene as a selectable marker between the 35S promoter and terminator (Figure 8a). Agrobacterium-mediated transformation was performed via the floral dipping technique of *Arabidopsis thaliana* (ecotype Columbia) [36]. Over-expressing transgenic plants were selected by growing seeds on MS/2 agar medium containing 50 mg/L kanamycin, which were grown up to the T3 generation to identify plants homozygous for the transgene.

### 4.5. Isolation of RNA, qRT-PCR

Total RNA was extracted from 200 mg of young leaves from *Arabidopsis transgenic* lines or silenced pepper plants using the RNeasy total RNA isolation kit (TianGen, Beijing, China). The cDNA was made by using PrimScript RT Kit (TaKaRa, Dalian, China). Primers are presented in Supplementary Table S1. The qRT-PCR was carried out as described previously [23]. The CaUbi3 gene (GenBank Accession No. AY486137.1) encoding the ubiquitin-conjugating protein was amplified from pepper plants as a reference gene for normalization of the *CaDHN5* cDNA samples [37], and the Atactin gene (GenBank Accession No. AY572427.1) was used as an internal control in *Arabidopsis* [38]. The relative fold difference in mRNA levels was determined using the 2^−ΔΔ*C*T^ method.

### 4.6. Measurement of Correlative Physiological Indices

#### 4.6.1. Determination of MDA Content

Approximately 0.5 g of pepper leaves were weighed and rapidly ground with pre-chilled 10% trichloroacetic acid solution. Finally, to the mixed solution, 10% trichloroacetic acid was added to reconstitute the solution to 10 mL, and centrifuged at 4000 rpm for 10 min at 4 °C. A volume of 2 mL supernatant was taken and mixed with 2 mL 0.6% thiobarbituric acid solution. The mixed solution was heated in boiling water for 15 min and rapidly cooled. Following centrifugation at 4000 rpm for 10 min at 4 °C, the absorbance of the supernatant was measured at 532 nm, 450 nm, and 600 nm, according to the method described previously [39].

#### 4.6.2. Total Chlorophyll Content

Pepper leaves (0.1 g) were immersed in 95% ethanol. After the leaves were completely decolored, the absorbance of the supernatant was measured at 470 nm, 649 nm, and 665 nm, as described previously [40].

#### 4.6.3. Relative Electrolyte Leakage

Electrolyte leakage was measured according to the method described previously [41]. Leaves from treated and control plants were selected; 10 leaf discs were made by using a perforator, and the leaf discs were placed in a 50 mL centrifuge tube containing 10 mL of distilled water. After being kept at room temperature for 2 h, electrolyte leakage (EC1) was measured. The centrifuge tubes were heated in boiling water for 30 min after cooling, and the conductivity measurement value (EC2) was measured. Relative electrolyte leakage was calculated as (EC1/EC2) × 100.

#### 4.6.4. Enzyme Activity

The SOD and POD activities were measured according to a previously described method [42]. Fresh leaves (0.5 g) were mixed with 8 mL PBS pH 7.8 and the mixture was centrifuged at 10,000 rpm for 15 min. The supernatant was considered as the crude enzyme extract. In the presence of hydrogen peroxide, POD can oxidize guaiacol to produce colored substances; the product concentration was calculated and POD activity was measured. SOD activity was determined by a similar principle, with NBT as the reaction substance.

#### 4.6.5. NBT Staining

The NBT staining method was as used as described previously [43]. The plant leaves were immersed in a 0.1 mg/L NBT solution in Tris-HCl, pH 7.8, and vacuum infiltrated for about 1 min. After being incubated for 1 h in the dark, the leaves were placed in 80% ethanol, which was changed twice. After complete removal of chlorophyll, the degree of leaf staining was observed.

#### 4.6.6. Water Loss Rate

Isolated plant leaves were placed on the laboratory bench (20–22 °C, humidity 45–60%) and their weight was measured every 30 min, as described previously [44]. The initial fresh weight of the leaves was recorded as W0, and thereafter weighed every 30 min. The leaf weight after 4 h was recorded as Wt. The water loss rate per 30 min was calculated as: (W0−Wt)/W0 × 100.

#### 4.6.7. Proline Content

Approximately 0.5 g leaves were mixed with 5 mL of 3% sulfosalicylic acid; the mixture was placed in a 100 °C water bath for 10 min. After cooling, the mixture was centrifuged at 3000 rpm for 10 min. The supernatant (extraction solution, 2 mL) was mixed with a color rendering agent, indene (2 mL), and glacial acetic acid (2 mL). The mixed solution was heated in boiling water for about 40 min. A volume of 5 mL toluene was added into the mixing solution after cooling and the absorbance value was measured at 520 nm, according to a previously described method [45].

### 4.7. Statistical Analysis

The qRT-PCR data analysis was carried out using SPSS (Chicago, IL, USA). The relative expression levels of CaDHN5 under salt and osmotic stress are shown as mean ± SD of three biological replicate samples. Each replicate sample was a composite of leaves from three individual seedlings. Statistical analyses were performed using the SPSS (Chicago, IL, USA), and the means were compared using Tukey’s HSD multiple range test, taking *p* < 0.05 as a significant difference.

## 5. Conclusions

In conclusion, although the physiological function of *CaDHN5* at a molecular level has not yet been identified, here we show that *Arabidopsis* plants overexpressing *CaDHN5* have higher survival rates in salt and osmotic stress conditions. These results suggest a functional role for *CaDHN5* in response to salt and osmotic stress. *Arabidopsis* plants overexpressing *CaDHN5* were significantly superior to WT in various physiological indices measured under salt and osmotic stresses. After gene silenced pepper plants, the tolerance of pepper plants to salt and mannitol were significantly decreased, and the above two factors jointly proved the effect of *CaDHN5* on plant tolerance to salt and osmotic stress.

## Supplementary Materials

Supplementary materials can be found at https://www.mdpi.com/1422-0067/20/8/1989/s1.
