# Supplementary material for: CaDHN5, a Dehydrin Gene from Pepper, Plays an Important Role in Salt and Osmotic Stress Responses
Source: Int J Mol Sci. 2019 Apr 23;20(8):1989. doi: 10.3390/ijms20081989 (PMC6514665; doi:10.3390/ijms20081989)
Supplement: Supplementary file 1 [file ijms-20-01989-s001.pdf]

**supplementary materials:**

**Table S1**. Primers.

| Gene | Primer | Sequence(5′-3′) |
|---|---|---|
| *AtActin* | *AtActin-F* | TGTTATGGTAGGGATGGGTC |
| | *AtActin-R* | TTCTCTCTATTTGCCTTGGG |
| *AtDREB2A* | *AtDREB2A-F* | GTGACCTAAATGGCGACGAT |
| | *AtDREB2A-R* | GCGGATCAAAACCACTTTGT |
| *AtDREB2B* | *AtDREB2B-F* | GAGAGAAACCGAAACGCAAAGTTCCTGC |
| | *AtDREB2B-R* | TAAGCGGAAGCAGCTTTTTCCGCGGTAG |
| *AtERD7* | *AtERD7-F* | ATCTCTTCCCTGAACAACCA |
| | *AtERD7-R* | GCGAGAACAGCGACAACAT 3' |
| *AtMYC2* | *AtMYC2-F* | CGATTGGAACACCTGGATCTAACG |
| | *AtMYC2-R* | GGGTTTTCGGTTATTGTGCTTGAG |
| *AtATR1/MYB34* | *AtATR1/MYB34-F* | GCGGGACGAACTGACAACGAAA |
| | *AtATR1/MYB34-R* | GCGGTTAAGAAGCCTAGCGGAA |
| *AtSOS1* | *AtSOS1-R* | TCGTTTCAGCCAAATCAGAAAGT |
| | *AtSOS1-R* | TTTGCCTTGTGCTGCTTTCC |
| *AtRITF1* | *AtRITF1-F* | CTCTGCTTCATCCGCCGCATCGTCTC |
| | *AtRITF1-R* | GAGCTCGATCGGCGGCTTCACGGACG |
| *AtRSA* | *AtRSA-F* | GAAGTTCAGTGAGTGGTGGCGCAGAAGGAG |
| | *AtRSA-R* | GGTGAATCAGGTAAAGTAGGGACATATC |
| *MnSOD* | *MnSOD-F* | CTCTGCCATAGACACCAACTT |
| | *MnSOD-R* | CCAAGTTCGGTCCTTTAATAA |
| *POD* | *POD-F* | GCAGCATTCCTCCTCCTACT |
| | *POD-F* | ATTTCTTTGCCTTGTTGTTG |
| *ERD15* | *ERD15-F* | CCAGCGAAATGGGGAAAC |
| | *ERD15-R* | ACAAAGGTACAGTGGTGGC |
| *CaUbi3* | *CaUbi3-F* | TGTCCATCTGCTCTCTGTTG |
| | *CaUbi3-R* | CACCCCAAGCACAATAAGAC |
